# Georgia State Dental Society

**Published:** 1875-08

**Authors:** Charles C. Allen

**Affiliations:** Cor. Secretary.


### ARTICLE III.

### *Georgia State Dental Society.*

The seventh annual meeting of this society was held in Atlanta, Monday, May 10th, adjourning May 13th. The President, Dr. Patterson, of Waynesboro', Ga., presiding.

The meeting was well attended, and gave evidence of much progress in the profession in Georgia. A flattering number of new members were added to the roll. The following reports were read by gentlemen composing the various committees:

*Chemistry and Therapeutics.*—Dr. E. Parsons, Savannah, Ga.

*Histology and Physiology.*—Dr. Charles C. Allen, Marietta, Ga.

*Operative Dentistry.*—Dr. A. C. Ford, Atlanta, and Dr. M. S. Jobson, Perry, Ga.

*Mechanical Dentistry.*—Dr. S. G. Holland, Atlanta, Ga.

*Dental Education.*—Dr. John H. Coyle, Thomasville, Ga

The address of the president, Dr. George Patterson, of Waynesboro', was an able and scientific production, well worthy of the applause it called forth. This address was ordered printed for the use of the members.

Dr. M. S. Jobson read a paper containing statistics of nerve-capping in his practice. It was a valuable addition to the archives of the society. Bearing upon the same subject was a very instructive essay by Dr. W. C. Wardlaw, of Augusta, advocating the use of the ordinary pattern plate gutta-percha dissolved in chloroform as a capping for exposed or aching pulps. Other essays were read by members of the society, and many valuable ideas presented.

Suitable resolutions were read and spread upon the minutes in regard to the death of Dr. J. A. Fuqua, of Atlanta, an esteemed and honored member of the Georgia State Dental Society.

One step taken, indicating a healthy ambition, was a resolution to make arrrangements for the purchase of a microscope for the use of the society.

On motion of Dr. E. Parsons, the president appointed a historian to collect all items of interest pertaining to the history of the society from its origin to the present time; to arrange in suitable form for publication the transactions, essays, debates, etc., and to report upon the same at the next meeting of the society.

The annual election resulted in the following list of officers to serve the society during the following year:

*President.*—Dr. George W. McElhany, West Point, Ga.

*First Vice-President.*—Dr. M. S. Jobson, Perry, Ga.

*Second Vice-President.*—Dr. John H. Coyle, Thomasville, Ga.

*Corresponding Secretary.*—Dr. Charles C. Allen, Marietta Ga.

*Recording Secretary.*—Dr. D. Smith, Atlanta, Ga.

*Executive Committee.*—Dr. E. Parsons, Savannah; Dr. W. C. Wardlaw, Augusta; Dr. A. C. Ford, Atlanta; Dr. W. F. Tigner, Columbus; Dr. John H. Coyle, Thomasville.

STANDING COMMITTEES.

*Histology and Physiology.*—Dr. George Patterson, Waynesboro', Ga; Dr. E. Parsons, Savannah,

*Pathology and Surgery.*—Dr. A. C. Ford, Atlanta; Dr. R. A. McDonald, Griffin.

*Chemistry and Therapeutics.*—Dr. J. D. McKellar, Macon; Dr. S. G. Holland, Atlanta.

*Operative Dentistry.*—Dr. S. G. Robertson, Cuthbert; Dr. W. C. Wardlaw, Augusta.

*Mechanical Dentistry.*—Dr. W. F. Tigner, Columbus; Dr. R. B. Adair, Gainesville.

*Dental Education and Literature.*—Dr. E. M. Allen, Marietta; Dr. H. A. Lowrance, Athens.

*Clinical Operators.*—Dr. L. D. Carpenter, Atlanta; Dr. James Hart, Hawkinsville.

*Historian.*—Dr. Charles C. Allen, Marietta.

*Committee on Instruments, Appliances, etc.*—Dr. L. D. Carpenter, Atlanta; Dr. S. G. Holland, Atlanta; Dr. J. P. Holmes, Macon.

*Committee on Microscope.*—Dr. A. C. Ford, and Dr. Samuel Hape, Atlanta.

*Committee on Arrangements.*— Dr. Samuel Hape and Dr. William Crenshaw, Atlanta.

*Delegates to American Dental Association.*—Dr. H. A Lowrance, Athens, Ga.; Dr. A. C. Ford, Atlanta, Ga.; Dr. J. H. Coyle, Thomasville, Ga.; Dr. M. S. Jobson, Perry, Ga.; Dr. Charles C. Allen, Marietta, Ga.

*Delegates to Southern Dental Association.*—Dr. E. M. Allen, Marietta, Ga.; Dr. J. P. Holmes, Macon, Ga.; Dr. W. F. Tigner, Columbus, Ga.; Dr. R. B. Adair, Gainesville, Ga.

Adjourned until the second Tuesday in May, 1876.

CHARLES C. ALLEN, D. D. S., *Cor. Secretary.*

